# The Stress Response Regulator AflSkn7 Influences Morphological Development, Stress Response, and Pathogenicity in the Fungus *Aspergillus flavus*

**DOI:** 10.3390/toxins8070202

**Published:** 2016-07-05

**Authors:** Feng Zhang, Gaopo Xu, Longpo Geng, Xiaoyan Lu, Kunlong Yang, Jun Yuan, Xinyi Nie, Zhenhong Zhuang, Shihua Wang

**Affiliations:** Key Laboratory of Pathogenic Fungi and Mycotoxins of Fujian Province, Key Laboratory of Biopesticide and Chemical Biology of the Education Ministry, and School of Life Sciences, Fujian Agriculture and Forestry University, Fuzhou 350002, China; fzhang@fafu.edu.cn (F.Z.); xugaopo99@outlook.com (G.X.); glpabc2016@outlook.com (L.G.); xylu6@outlook.com (X.L.); ykl_long@outlook.com (K.Y.); yuansunyuan@outlook.com (J.Y.); xinyi_nie@126.com (X.N.); xzhzhenhong@gmail.com (Z.Z.)

**Keywords:** *Aspergillus flavus*, aflatoxins, development, stress response, pathogenicity

## Abstract

This study focused on AflSkn7, which is a stress response regulator in the aflatoxin-producing *Aspergillus flavus*. The Δ*AflSkn7* mutants exhibited partially defective conidial formation and a complete inability to generate sclerotia, indicating AflSkn7 affects *A. flavus* asexual and sexual development. The mutants tolerated osmotic stress but were partially susceptible to the effects of cell wall stress. Additionally, the Δ*AflSkn7* mutants were especially sensitive to oxidative stress. These observations confirmed that AflSkn7 influences oxidative stress responses rather than osmotic stress responses. Additionally, AflSkn7 was observed to increase aflatoxin biosynthesis and seed infection rates. These results indicate AflSkn7 affects *A. flavus* morphological development, stress response, aflatoxin production, and pathogenicity. The results of this study may facilitate the development of new methods to manage *A. flavus* infections.

## 1. Introduction

*Aspergillus flavus*, a saprophytic soil fungus, causes preharvest and postharvest diseases in seed crops such as maize, peanut, and cottonseed [1]. This fungus produces the mycotoxin aflatoxin, which can affect the structure of DNA [2]. If animals or humans ingest aflatoxin-contaminated seeds, they may develop liver tumors [1]. Consequently, contamination of agricultural commodities by aflatoxin may result in considerable economic losses and serious health problems, especially in developing countries [2]. Aflatoxins are a group of toxic secondary metabolites that are mainly produced by two *Aspergillus* species (Section Flavi), namely *A. flavus* and *A. parasiticus* [3]. *Aspergillus flavus* can produce two aflatoxins, B_1_ and B_2_. Aflatoxin B_1_ (AFB_1_) is considered the most toxic aflatoxin, and its AFB_1_ epoxides and AFB_1_-exo-epoxides are often detected in hepatocellular carcinoma patients [1].

Previous studies on *A. flavus* revealed that approximately 30 genes clustered within a 70-kb genomic region near the telomere of chromosome 3 are involved in aflatoxin biosynthesis [1,4]. Most of these genes encode enzymes catalyzing one or multiple reactions in the aflatoxin biosynthetic pathway, while other genes encode regulator proteins. For example, *aflR* encodes a global regulatory protein required for transcriptional activation of most structural genes [5]. Additionally, many environmental factors regulate aflatoxin biosynthesis, including temperature, water activity, and oxidative stress [6,7,8,9]. Despite the crucial roles of environmental factors during aflatoxin biosynthesis, how aflatoxin producers sense and cope with various environmental stresses is currently unknown. Therefore, characterizing the underlying mechanism may enable the development of improved methods to control *A. flavus* infections.

Histidine kinase-mediated phosphorelays, also known as two-component signaling systems, are common fungal pathways for sensing and adapting to environmental changes. The Sln1 pathway in *Saccharomyces cerevisiae* is one of the best-studied two-component pathways, and it primarily responds to osmotic stress [10]. Skn7 (Suppressor of *kre* nine 7) is responsible for another branch of the Sln1 pathway. In *S. cerevisiae*, Skn7 functions in response to oxidative and cell wall stresses using different mechanisms [11]. Following exposure to cell wall stress, the conserved aspartate residue of Skn7 is phosphorylated via Ypd1. In contrast, Skn7 is phosphorylated at a conserved threonine residue in the receiver domain in response to oxidative stress [12]. However, one study revealed that Skn7 is also involved in responses to osmotic stress [13]. Furthermore, there is considerable evidence linking Skn7 function to thermal stress adaptation, fungicide sensitivity, sexual mating, autolysis, sporulation, and virulence in other fungi [10,14].

Therefore, the main objective of this study was to characterize *A. flavu*s Skn7 functions regarding morphological development, stress responses, and pathogenicity. A thorough characterization of Skn7 functions may facilitate the development of new methods to control *A. flavus* infections. Δ*AflSkn7* deletion mutants were generated, and the mutant phenotypes related to stress and virulence were analyzed.

## 2. Results

### 2.1. Aspergillus Flavus Skn7 Sequence Analysis

**Figure 1:**
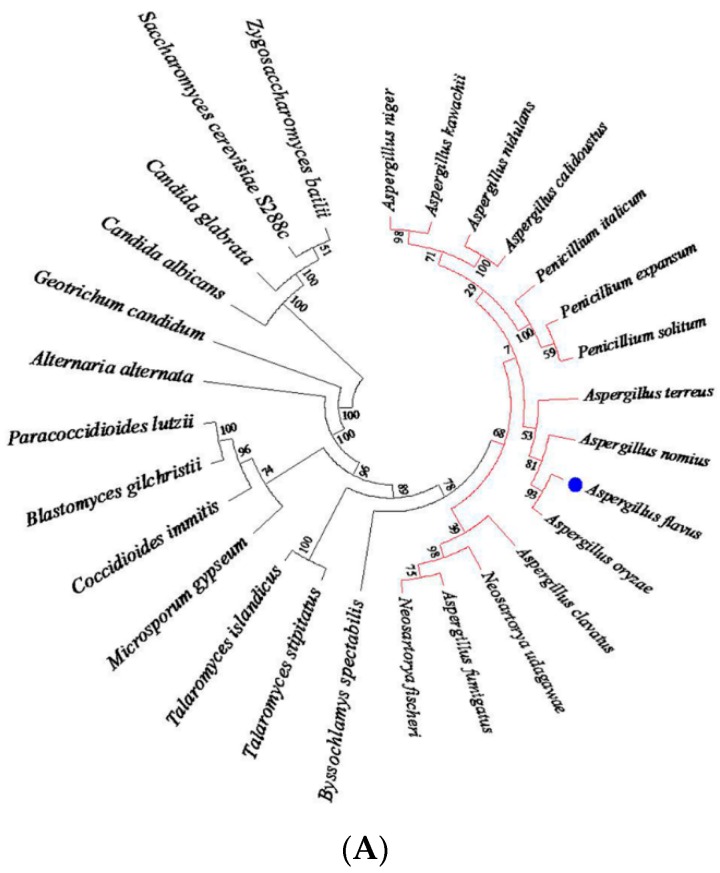

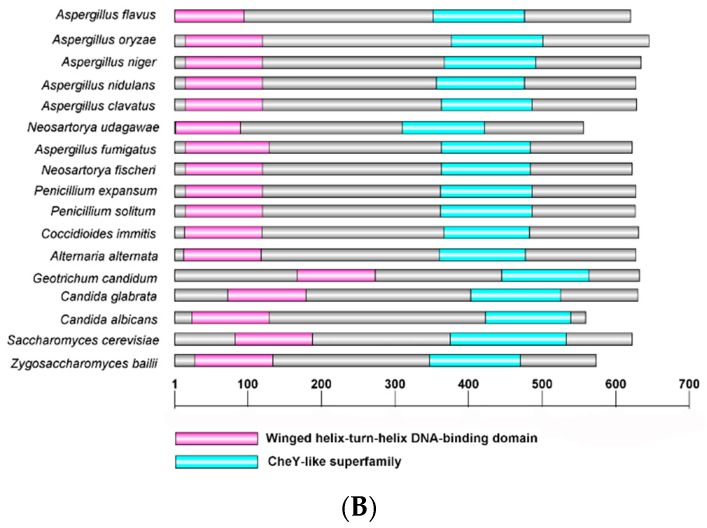
Sequence and domain analysis of Skn7 proteins from different fungi. (**A**) Phylogenetic analysis of Skn7 proteins from 28 fungal species. Bootstrap values were calculated using the neighbor-joining method with 1000 replicates and are indicated at each node. All clades with *Aspergillus* species are highlighted in red, and *Aspergillus flavus* is indicated in blue point. (**B**) Schematic diagrams of Skn7 proteins from different fungi. Gray parts correspond to non-conserved regions. Pink parts indicate the heat-shock transcription factor-like DNA-binding domain. Green parts represent the CheY-like receiver domain. The scale corresponds to protein length.

The sequence of the *A. flavus* putative response regulator *Skn7* (AFL2G_02624) was retrieved from the Broad Institute database. Based on the annotation, the *AflSkn7* open reading frame (ORF) consists of 2851 bp and is interrupted by four introns (76, 60, 62, and 59 bp). The encoded amino acid sequence is highly similar to orthologous sequences from other fungi, and is most similar to *Aspergillus oryzae* Skn7 (Figure 1A). Sequence analysis revealed that AflSkn7 contains two conserved domains: an *N*-terminal heat-shock transcription factor-like DNA-binding domain and a *C*-terminal CheY-like receiver domain (Figure 1B). These two domains are common among Skn7-like proteins from other fungi, suggesting AflSkn7 regulates stress responses in *A. flavus*.

### 2.2. Construction of ΔAflSkn7 Deletion Mutants

**Figure 2:**
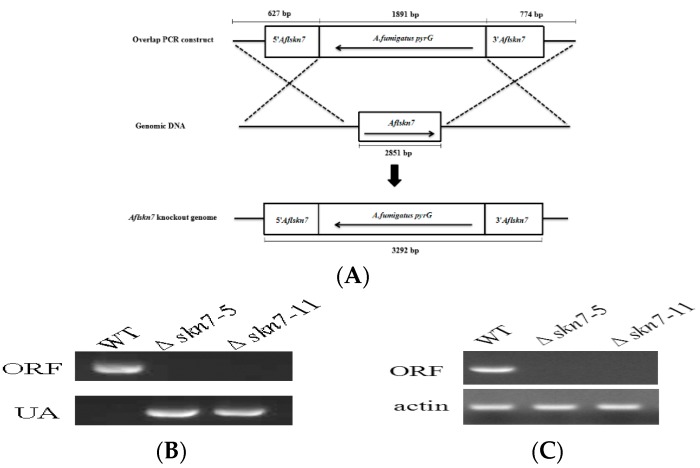
Gene deletion method and confirmation. (**A**) Schematic illustration of the gene deletion method. The numbers indicate the length of the corresponding gene. (**B**) Polymerase chain reaction (PCR) confirmation of Δ*AflSkn7* gene deletion. ORF, *AflSkn7* coding region amplified by PCR; UA, 5′ region of *AflSkn7* amplified by PCR. (**C**) Reverse transcription-PCR confirmation of Δ*AflSkn7* gene deletion.

To confirm the biological function of Skn7 in *A. flavus*, Δ*AflSkn7* deletion mutants were constructed using a homologous recombination strategy (Figure 2A). Knockout mutants were screened from uridine autotrophy transformants by polymerase chain reaction (PCR) analysis. Two deletion mutants (i.e., *skn7-5* and *skn7-11*) were identified with two PCR primer pairs, which amplified fragments that corresponded to the ORF and 5′ flanking regions of *AflSkn7*, respectively (Figure 2B). To avoid false positives, PCR primers (i.e., AflSkn7UUF/AflSkn7DDR) matched to the upstream and downstream regions of fusion fragment were used to verify the transformants. When PCR products were sequenced, the alignment results indicated 100% identity between the sequenced products and the expected sequences (Figure S1). The PCR and sequencing results indicated that *AflSkn7* was exactly replaced by *pryG* in knockout mutants. Additionally, reverse transcription (RT)-PCR using primers specific for the ORF region confirmed *AflSkn7* was not expressed in deletion mutants (Figure 2C), suggesting *AflSkn7* gene was disrupted in *skn7-5* and *skn7-11* strains. Furthermore, the stable and identical phenotype of the two mutants as following suggested that changes in these mutants were caused by the inactivation of *AflSkn7* in *A. flavus*.

### 2.3. Involvement of AflSkn7 during Asexual Development

**Figure 3:**
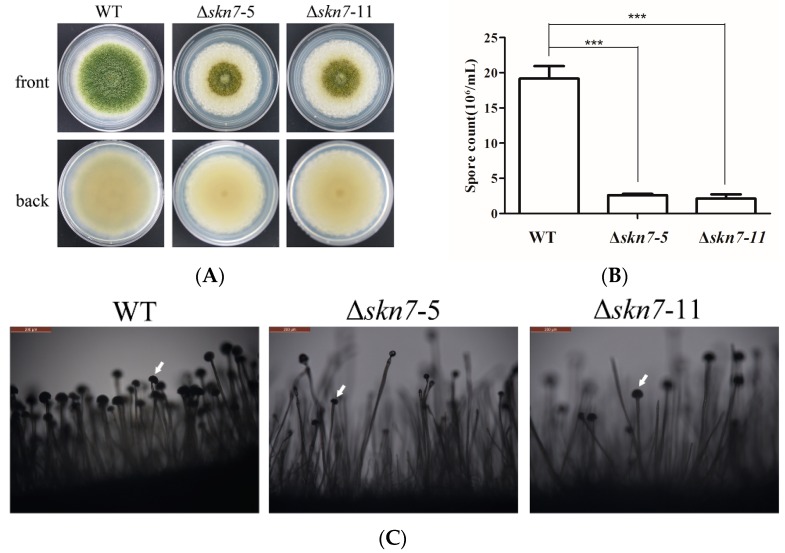
Effects of *AflSkn7* on conidial formation: (**A**) colony morphology of strains during conidiation; (**B**) comparison of conidial production among wild-type fungus and two mutants; and (**C**) microscopic analysis of conidial structures (magnification: ×200). Conidiophores for each strain are indicated by white arrows. All strains were cultured on potato dextrose agar medium at 28 °C for seven days. Asterisks indicate significant differences from the results of the wild-type fungus (*** *p* ≤ 0.001).

The growth rates of the wild-type fungus and Δ*AflSkn7* deletion mutants were similar on potato dextrose agar (PDA) medium at 28 °C (Figure 3A). In contrast, there were considerable differences in the production of conidia on PDA medium (Figure 3A), with the wild-type fungus producing approximately five-fold more conidia than the mutants (Figure 3B). Microscopic examinations indicated Δ*AflSkn7* mutants produced fewer conidiophores and less aerial hyphae than the wild-type fungus (Figure 3C). Additionally, the mutant conidiophores were smaller than those of the wild-type fungus, suggesting the loss of *Skn7* might delay conidiation. These findings suggested AflSkn7 was involved in *A. flavus* asexual development.

### 2.4. Requirement of AflSkn7 during Sexual Development

**Figure 4:**
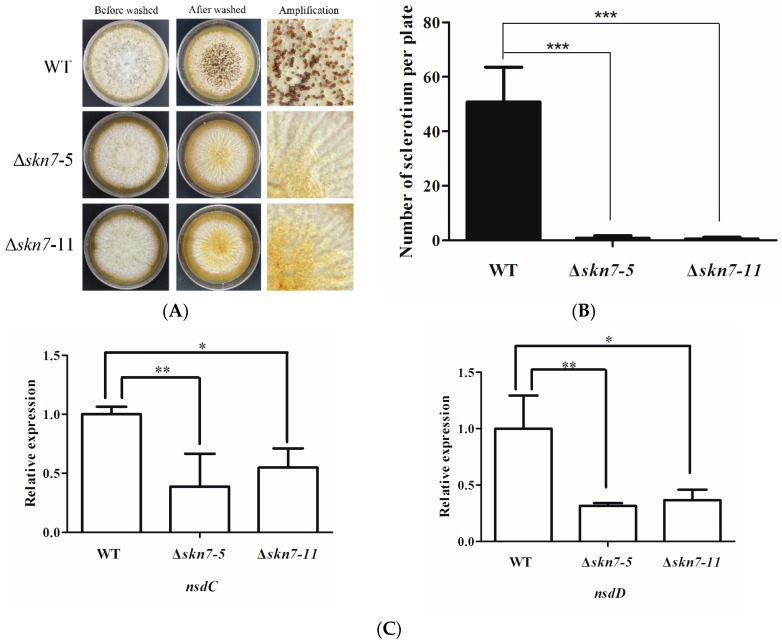
Effects of AflSkn7 on sclerotial formation. (**A**) Colony morphology of strains with sclerotia. Enlarged image of each plate is provided on the right side. (**B**) Abundance of sclerotia produced by the wild-type fungus and two mutants. (**C**) Expression analysis of two genes related to sclerotial formation in the wild-type fungus and two mutants. All strains were cultured on YES medium at 37 °C. After incubating for seven days, cultures were washed with 75% ethanol. The 2^−ΔΔCt^ method was used to evaluate of target gene expression levels, which were relative to that of the β-actin reference gene. Asterisks indicate significant differences from the results of the wild-type fungus (* *p* ≤ 0.05; ** *p* ≤ 0.01; *** *p* ≤ 0.001).

Considering it affects asexual development, the potential roles of AflSkn7 during sexual development were investigated. After culturing fungal strains on yeast extract-sucrose (YES) medium at 37 °C for seven days, all strains were washed with 75% ethanol. There were almost no morphological differences between the wild-type and mutant strains prior to the ethanol wash (Figure 4A, left). After the wash, the wild-type fungus was able to produce sclerotia on YES medium (Figure 4A, top), while both Δ*AflSkn7* mutants exhibited severely limited sclerotial production (Figure 4A, middle and bottom). A quantitative analysis revealed that the Δ*AflSkn7* mutants produced far fewer than the wild-type strain (Figure 4B). A quantitative RT-PCR (qRT-PCR) method was used to analyze the expression of *nsdC* and *nsdD*, which influence sclerotial development. The *nsdC* expression level in the wild-type fungus was at least two-fold higher than that of the Δ*AflSkn7-5* mutant (Figure 4C, left). Additionally, the *nsdD* expression level was at least four-fold higher in the wild-type strain than in the Δ*AflSkn7-5* mutant (Figure 4C, right). These data indicated that *AflSkn7* had a considerable effect on *A. flavus* sexual development.

### 2.5. Effects of the Deletion of AflSkn7 on Sensitivity to Osmotic and Cell Wall Stresses

**Figure 5:**
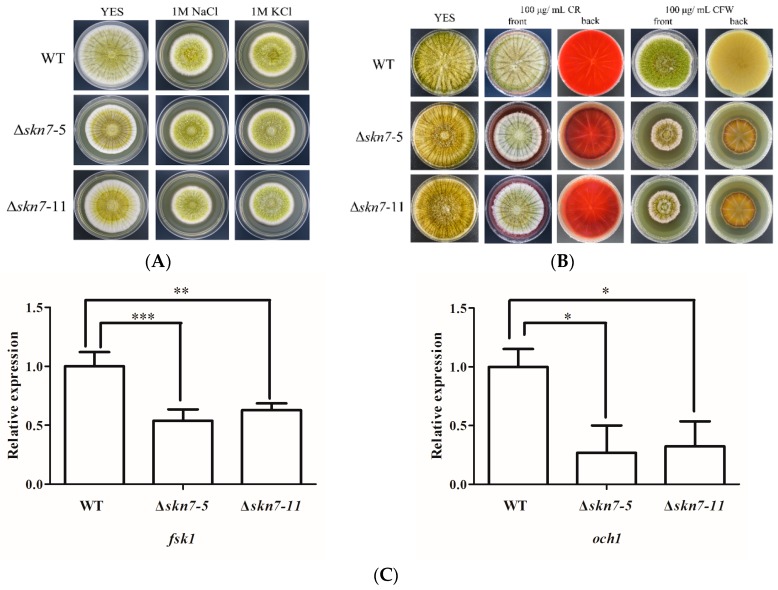
Effects of osmotic and cell wall stresses on wild-type (WT) fungus and two mutants (Δ*Skn7-5* and Δ*Skn7-11*): (**A**) colony morphology of strains under different salt stress conditions; (**B**) colony morphology of strains treated with different cell wall damaging agents; and (**C**) expression analysis of two genes related to cell wall synthesis in WT and mutant fungi. The 2^−ΔΔCt^ method was used to evaluate target gene expression levels, which were relative to the expression level of the β-actin reference gene. Asterisks indicate significant differences from the results of the wild-type fungus (* *p* ≤ 0.05; ** *p* ≤ 0.01; *** *p* ≤ 0.001).

Skn7 responds to osmotic and cell wall stresses in many fungi. In this study, the sensitivity of *A. flavus* to different osmoticants and cell wall-damaging agents was examined. Under osmotic stress conditions, the mycelial growth rate of all *A. flavus* strains decreased. However, disruption of the *AflSkn7* gene did not result in increased sensitivity to NaCl and KCl (Figure 5A). To analyze the sensitivity of the Δ*AflSkn7* mutants to cell wall stress, all strains were grown on YES medium supplemented with Congo red and calcofluor white. Both agents subtly affected the radial growth of the wild-type fungus (Figure 5B). In contrast, both agents strongly inhibited the growth of Δ*AflSkn7* mutants. Furthermore, the adverse effects caused by calcofluor white were greater than those of Congo red (Figure 5B). The expression levels of *fsk1* and *och1*, which are cell wall synthesis genes, were significantly difference between the wild-type fungus and the mutants, suggesting the cell wall components of the ΔAfl*Skn7* mutants were disordered (Figure 5C). Therefore, AflSkn7 responds to cell wall stress, but not to osmotic stress in *A. flavus*.

### 2.6. Effects of the Deletion of AflSkn7 on Sensitivity to Oxidative Stress

**Figure 6:**
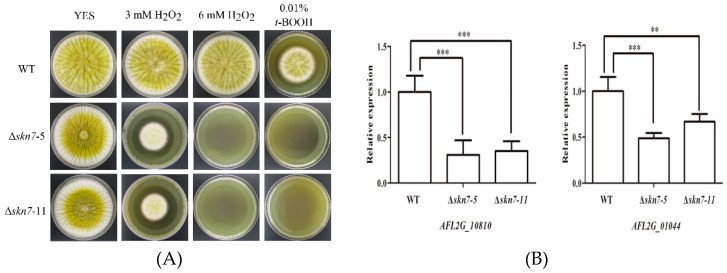
Effects of oxidative stress on wild-type (WT) fungus and two mutants (Δ*Skn7-5* and Δ*Skn7-11*): (**A**) colony morphology of strains exposed to different oxidative stresses; and (**B**) quantitative reverse transcription polymerase chain reaction analysis of antioxidant gene expression. The 2^−ΔΔCt^ method was used to evaluate of target gene expression levels, which were relative to that of the β-actin reference gene. Asterisks indicate significant differences from the results of the wild-type fungus (** *p* ≤ 0.01; *** *p* ≤ 0.001).

Oxidative stress is another important environmental stimulus for fungi. There is abundant evidence indicating the secondary metabolism and pathogenicity of filamentous fungi are affected by oxidative stress [15]. In this study, *A. flavus* strains lacking *AflSkn7* were hypersensitive to hydrogen peroxide (H_2_O_2_) and *tert*-butyl-hydrogen peroxide (*t*-BOOH) (Figure 6A). Mutant growth was completely inhibited by 6 mM H_2_O_2_. In contrast, growth of the wild-type fungus was unaffected even at 6 mM H_2_O_2_ (Figure 6B). The expression levels of two genes encoding antioxidants (i.e., Cu, Zn-superoxide dismutase: AFLG_10810 and glutathione reductase: AFLG_01044) were higher in the wild-type fungus than in the two mutants (Figure 6C). The expression level differences between the wild-type and mutant strains were greater for the superoxide dismutase gene than for the glutathione reductase gene. These observations implied that AflSkn7 has a critical role during responses to oxidative stress.

### 2.7. Role of AflSkn7 for Aflatoxin Biosynthesis

**Figure 7:**
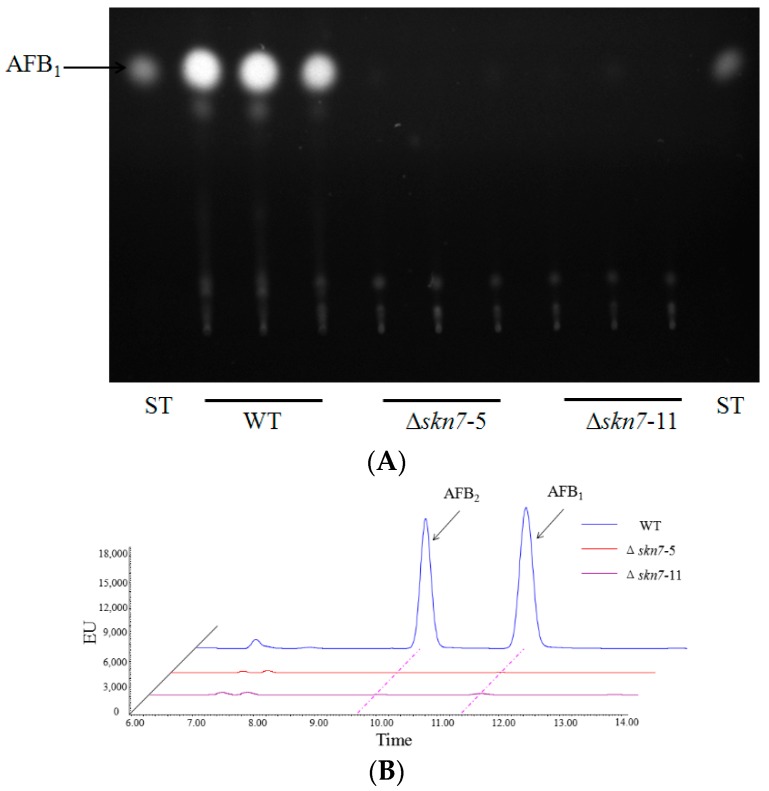

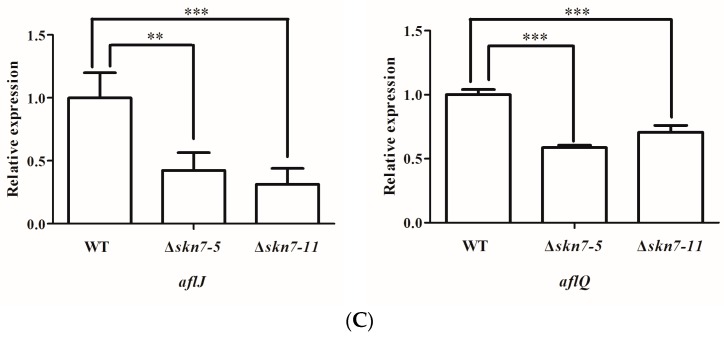
Roles of AflSkn7 for aflatoxin biosynthesis: (**A**) thin-layer chromatography analysis of aflatoxin production in the indicated strains; (**B**) high-performance liquid chromatography analysis of aflatoxin production in the indicated strains; and (**C**) quantitative reverse transcription polymerase chain reaction analysis of the expression levels of two aflatoxin biosynthesis genes. The 2^−ΔΔCt^ method was used to evaluate target gene expression levels, which were relative to the expression level of the β-actin reference gene. Asterisks indicate significant differences from the results of the wild-type fungus (** *p* ≤ 0.01; *** *p* ≤ 0.001).

As previously mentioned, the virulence of *A. flavus* is partially based on the production of aflatoxins. Therefore, the role of AflSkn7 for aflatoxin biosynthesis was investigated. Only the wild-type *A. flavus* strain produced aflatoxins in YES broth at 28 °C (Figure 7A). This was consistent with the results of strains cultured in potato dextrose broth (PDB) broth (Figure S2). Quantitative analyses of both Δ*AflSkn7* mutants indicated they were unable to produce aflatoxins in YES broth (Figure 7B). To confirm this observation, the expression levels of two aflatoxin biosynthesis genes (*aflJ* and *afl*Q) were analyzed in all strains. As expected, the transcription levels of the aflatoxin biosynthesis genes in the Δ*AflSkn7* mutants were considerably lower than that in the wild-type strain (Figure 7C). All evidence indicated that AflSkn7 increased aflatoxin biosynthesis in *A. flavus*.

### 2.8. Contribution of AflSkn7 to Pathogenicity

**Figure 8:**
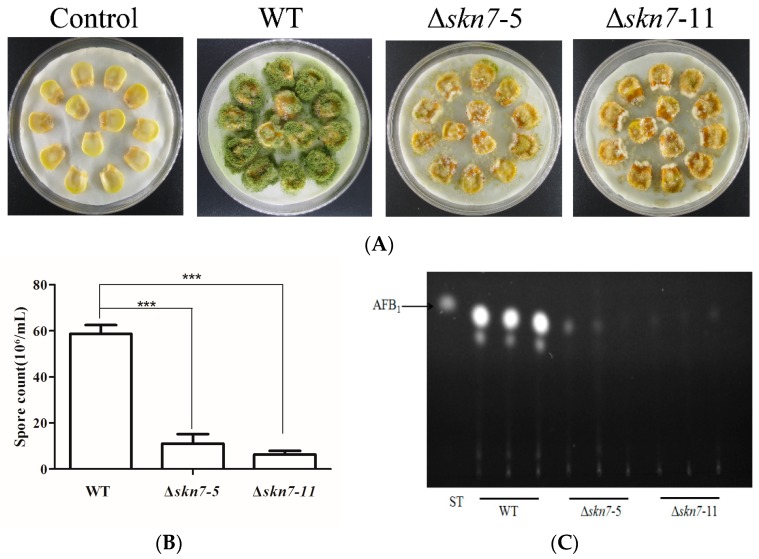
Effects of deletion of *AflSkn7* on fungal pathogenicity on maize: (**A**) maize kernels were treated with water (control), wild-type fungus (WT), or two *AflSkn7* deletion mutants (Δ*Skn7-5* and Δ*Skn7-11*) and cultured at 28 °C for seven days; (**B**) quantitative analysis of conidia collected from infected maize kernels; and (**C**) thin-layer chromatography analysis of aflatoxin production in infected maize kernels. Asterisks indicate significant differences from the results of the wild-type fungus (*** *p* ≤ 0.001).

Whether AflSkn7 affects the ability of *A. flavus* to colonize seeds and produce aflatoxin was investigated. The wild-type and mutant strains were still able to infect maize kernels seven days after inoculation. However, the wild-type fungus grew better on kernels than the Δ*AflSkn7* mutants, and also produced more conidia (Figure 8A,B). To examine the consequences of fungal infections of seeds, aflatoxin levels on contaminated seeds were determined. Similar to the results observed for the fungal strains grown in YES broth, the wild-type fungal strain produced more aflatoxin on infected kernels than the Δ*AflSkn7* mutants (Figure 8C). These results indicated AflSkn7 affected not only *A. flavus* colonization, but also aflatoxin biosynthesis. Thus, AflSkn7 likely influences *A. flavus* pathogenicity.

## 3. Discussion

Skn7 and its orthologs are widely distributed in fungi, suggesting they have important roles influencing fungal survival. The results presented herein are consistent with those of a previous study, which reported that an *Aspergillus nidulans Skn7* mutant (i.e., Δ*SrrA*) exhibits partly defective conidiation [16]. Another study confirmed that *SrrA* considerably affects the expression of *brlA* during conidial formation [17]. In *Alternaria alternata*, the defective conidia observed in *Skn7* mutants may be the result of inhibited cell wall biosynthesis [18]. In contrast to the results of the current study, the *Metarhizium robertsii* *Skn7* mutant is completely unable to produce conidia [14], while *A*. *fumigatus Skn7* is likely not involved in regulating the production of conidia [19]. These results indicate that Skn7 regulates conidial formation in a species-specific manner.

In addition to the effects of Skn7 on the production of conidia, this study determined that Afl*Skn7* positively influences the formation of sclerotia, suggesting Skn7 facilitates *A. flavus* sexual development. However, Skn7 did not affect sexual development in *Fusarium graminearum* [20], while *Cryptococcus neoformans* Skn7 inhibited sexual reproduction [21]. Interestingly, a *Botrytis cinerea Skn7* mutant produced more sclerotia than the wild-type fungus, although they were smaller [22]. Additionally, similar to the results of this study, the *Schizosaccharomyces pombe* Skn7 (i.e., Prr1) positively affected sexual development [23]. In *S. pombe*, Prr1 induces *Ste11* expression, which is essential for sexual development [23]. In *A. nidulans*, the *SteC* gene, which is a homolog of *S. pombe Ste11*, is important for sexual development [24]. There is currently no conclusive evidence that Skn7 regulates sexual development in the model filamentous fungus *A. nidulans*.

An important function of Skn7 in *A. flavus* and other fungi is its response to oxidative stress [17]. Wild-type *A. nidulans* can tolerate up to 2 mM H_2_O_2_, while the Δ*srrA* mutants are unable to grow in the presence of 1 mM H_2_O_2_ [16]. Similarly, the *A. fumigatus Skn7* mutant is hypersensitive to oxidative stress mediated by H_2_O_2_ [19]. Skn7 is believed to induce the expression of several antioxidant genes that provide resistance to H_2_O_2_, including genes for catalase, superoxide dismutase, and thioredoxin [16,25]. These results are consistent with the observations described herein, which indicated that Δ*AflSkn7* mutants are hypersensitive to oxidative stress and exhibit decreased antioxidant gene expression levels. In contrast with the findings of the studies mentioned above, in the insect-pathogenic fungus *Metarhizium robertsii*, Skn7 did not influence responses to oxidative stress [14]. Likewise, in the rice pathogen *Magnaporthe oryzae*, sensitivity to oxidative stress was not altered in *Skn7* mutants, suggesting other signal transduction systems may be involved in responses to oxidative stress [26].

A role for Skn7 in responses to cell wall stress was also noted. The Δ*AflSkn7* mutants were more sensitive to cell wall damaging agents than the wild-type fungus. Congo red and calcofluor white decrease β-glucan levels and chitin accumulation in cell walls, respectively [17]. In Δ*MrSkn7* mutants, the accumulation of glucan and chitin components is inhibited. Additionally, the expression levels of genes associated with cell wall biosynthesis are significantly lower in the mutants than in the wild-type fungus [14]. Genes that regulate cell wall biosynthesis usually play a role in cell wall integration (CWI) signaling. The fact that Skn7 interacts with guanosine triphosphate (GTP)-bound Rho1, which is considered to be the main regulator of CWI signaling in yeast, strongly suggests Skn7 has a role in CWI signaling [27]. Therefore, the Rho1-mediated Slt2 mitogen-activated protein kinase pathway may influence AflSkn7 responses to cell wall stress. Additionally, the *ChAP1*/*Skn7 Cochliobolus heterostrophus* double mutant is more sensitive to cell wall stress than either of the single mutants [11]. Thus, Δ*AflSkn7* mutants may become more sensitive to wall stress following the deletion of *AflAP1*. The current study revealed that *A. flavus* Skn7 mutants were insensitive to osmotic stress. However, the radial growth of the *A. nidulans ΔsrrA* mutant decreased slightly following exposure to osmotic stress conditions [16]. It is possible that the stress response regulators Skn7 and Ssk1 may periodically have overlapping roles [10]. The overlapping functions of Skn7 and Ssk1 have been observed in *M. oryzae* [26]. Additional research is required to determine the exact relationship between Skn7 and Ssk1 in *A. flavus.*

Aflatoxin biosynthesis in filamentous fungi is induced and intensified by intracellular oxidative stress [15]. However, Roze and co-workers reported that aflatoxin biosynthesis resulted in the production of secondary reactive oxygen species (ROS) potentially able to provide resistance to oxidative stress [28]. In *A. parasiticus*, *ApyapA* (a yeast *yap1* homolog) controls the expression levels of several oxidative stress response genes involved in scavenging ROS [29]. The disruption of *ApyapA* led to a precocious ROS formation and aflatoxin biosynthesis [29]. Similar results were observed in *A. ochracus*, in which ochratoxin A biosynthesis is regulated by the *yap1*-homolog Ao*yap1* under oxidative stress condition [30]. Additionally, Skn7 is associated with Yap1 in yeast responses oxidative stress [12], and Hagiwara and co-workers proposed that *SrrA* and *NapA* (*yap1* homolog) formed a complex to activate antioxidant genes in *A. nidulans* [17]. A study on the promoters of aflatoxin genes and stress–response genes revealed that the SrrA and yap1 binding-sites were located close together in the promoters [31]. Therefore, SrrA may interact indirectly with Apyap1 to activate the expression of aflatoxin genes. The current study has for the first time provided direct evidence that Skn7 is very important for regulating aflatoxin biosynthesis. As complementary evidence, deoxynivalenol production by *F. graminearum* is lower in *FgSkn7* mutant strains than in wild-type fungus [20]. Furthermore, deoxynivalenol biosynthesis is upregulated when *F. graminearum* is exposed to oxidative stress [20].

The effect of Skn7 on pathogenicity is highly variable among plant pathogens. The pathogenicity of an *M. oryzae Skn7* mutant on rice is similar to that of the wild-type strain [26], and the *FgSkn7* mutant maintains full virulence on wheat [20]. However, deleting *Skn7* from *A. alternata* significantly influences virulence, and the contribution of *AaSkn7* on pathogenicity is closely linked to fungal antioxidant activities [18]. The ability of pathogens to overcome oxidative stress conditions created by a plant host is critical for successful colonization [32]. This is supported by the fact the Δ*AflSkn7* mutants analyzed in this study simultaneously inhibited antioxidant synthesis and colonization of maize (Figure 6B and Figure 8A). The efficiency of antioxidative activities is unexpectedly significantly decreased in a *B. cinerea Skn7* mutant, but the pathogen is just as virulent on tomato and cucumber leaves as the wild-type strain [13]. However, pathogenic fungi must overcome several obstacles, including osmotic stress and wall stress, to colonize in plant hosts. For example, *M. oryzae* must adapt to osmotic stress during infection [26]. Thus, the inhibited ability of Δ*AflSkn7* mutants to adapt to cell wall stress may restrict their ability to infect plants. The Δ*AflSkn7* mutants produced little aflatoxin and were weakly pathogenic on maize kernels (Figure 8A,C). A low aflatoxin producer usually exhibits a low germination rate, which may also influence the pathogenicity of a mutant [28]. In summary, the roles of Skn7 during development, aflatoxin production, adaptation to environmental stresses, and resistance to host defense mechanisms may affect pathogen virulence [1].

## 4. Materials and Methods

### 4.1. Fungal Strains and Growth Conditions

The *Aspergillus flavus* CA14 wild-type (Δ*ku70,* Δ*niaD*) and recipient (Δ*ku70*, Δ*pyrG,* Δ*niaD*) strains were obtained from Dr. Ana M. Calvo, Northern Illinois University, DeKalb, IL, USA. The fungi were cultured on YES medium (20 g yeast extract, 150 g sucrose, 1 g MgSO_4_·7H_2_O, and 1 L water) at 28 °C. Fungal strains were grown on PDA (Difco) under light for 5 days to produce conidia. Fungi were grown in yeast glucose trace element (YGT) broth (5 g yeast extract, 20 g glucose, 1 mL trace elements, and 1 L water) at 37 °C on a rotary shaker for mycelial growth, and strains were grown on potato dextrose agar (YPD) medium (10 g yeast extract, 20 g peptone, 20 g glucose, 20 g agar, and 1 L water) to produce sclerotia. Czapek-Dox medium (Difco) supplemented with 3% sucrose was used for mutant selection.

### 4.2. Sequence Analysis

The *AflSkn7* (AFL2G_02624) sequence was retrieved from the Broad Institute database [33] Sequences of other *Skn7* orthologs were downloaded from the National Center for Biotechnology Information database using the BLASTP algorithm with the AflSkn7 protein sequence as a query. Domains were predicted using the Simple Modular Architecture Research Tool [34] and drawn with the Illustrator for Biological Sequences program [35]. Sequences were aligned using ClustalW, and a phylogenetic tree was generated with MEGA5.1 (Center for Evolutionary Medicine and Informatics, Tempe, AZ, USA, 2012).

### 4.3. Gene Deletion

Gene knockout mutants were generated using homologous recombination [36]. For the homologous fragments, the 5′ and 3′ regions of *AflSkn7* (921 and 1043 bp, respectively) were amplified with the primer pairs AflSkn7UF/AflSkn7UR and AflSkn7DF/AflSkn7DR (Table S1), which contain sequences that overlap the marker gene. For the marker gene, *pyrG* was amplified with the primer pair pyrGF/pyrGR (Table S1). The resulting PCR products were purified and linked by overlapping PCR with the primer pair AflSkn7UF/AflSkn7DR. The fusion PCR products were purified and inserted into recipient strain protoplasts using a published procedure [33]. Putative transformants were verified by diagnostic PCR (using primer pairs AflSkn7UF/pyrGR and AflSkn7F/AflSkn7R) and RT-PCR (using primer pairs AflSkn7RTF and AflSkn7RTR) (Table S1). Putative mutants were further verified with one PCR primer pair (AflSkn7UUF/AflSkn7DDR), and PCR products were sequenced.

### 4.4. Molecular Manipulations

Fungal genomic DNA was extracted from mycelia grown in YGT broth at 37 °C for 24 h with shaking (180 rpm). Genomic DNA was isolated using lysis buffer (400 mM Tris-HCl, 60 mM EDTA, 150 mM NaCl, and 1% SDS). Briefly, mycelia were harvested, mixed with 500 μL lysis buffer, and incubated at 60 °C for 20 min. Potassium acetate (200 μL) was added to the mixture. After gentle mixing, the sample was centrifuged at 12,000× g for 10 min. The supernatant was gently mixed with 500 μL isopropanol (i.e., slow inversions). The resulting DNA pellet was collected by centrifugation at 12,000× g for 2 min. The pelleted DNA was washed with 1 mL 70% ethanol, air-dried for 5 min, and suspended in 30 μL TE buffer (400 mM Tris-HCl and 60 mM EDTA). TRIzol reagent (Bioteck, Beijing, China) was used to isolate RNA according to the manufacturer’s instructions.

### 4.5. Stress Sensitivity Assay

Conidia from each fungal strain were cultured on PDA under light at 37 °C for 5 days. Conidia were harvested by washing the PDA surface with sterile distilled water containing 0.05% Tween 20 and 7% DMSO (Sinopharm Chemical Reagent, Shanghai, China), and then counted using a hemocytometer. A conidial suspension (10^4^) was used to inoculate YES medium supplemented with 100 μg/mL calcofluor white (Sigma, St. Louis, MO, USA), 100 μg/mL Congo red (Sigma, St. Louis, MO, USA), 1 M NaCl, or 1 M KCl. Different concentrations of H_2_O_2_ were added to the YES medium for the oxidative stress assay. All plates were incubated in darkness at 37 °C for 5 days, and colonies were photographed.

### 4.6. Aflatoxin Analysis

Approximately 10^6^ conidia of each strain were used to inoculate 25 mL YES broth or PDB (supplemented with zinc and molybdenum ions) in 100-mL flasks, which were then incubated at 28 °C with shaking at 180 rpm. After 6 days, 25 mL chloroform was added to each flask, and cultures were incubated for an additional 1 h. A 10-mL aliquot from the chloroform layer was transferred to a clean tube and evaporated to dryness at 70 °C. The solid residue was resuspended in 100 μL chloroform, and a 20-μL sample was spotted onto a thin-layer chromatography plate (Si250F; Jindao, Qingdao, China). The plate was then treated with a chloroform:acetone (95:5, *v*/*v*) solution and viewed under UV light. Aflatoxins were quantified using a high-performance liquid chromatography system with a UV light director. A solid residue sample was resuspended in methanol, and a 15-μL suspension was injected into a reversed-phase (C-18 column) high-performance liquid chromatography system (Waters, Milford, MA, USA). The solvent consisted of water:methanol:acetonitrile (56:22:22, *v*/*v*/*v*) at a flow rate of 1 mL/min.

### 4.7. Quantitative Reverse Transcription Polymerase Chain Reaction Analysis

Extracted RNA (2 µg) was treated with DNase I (Thermo Fisher Scientific, Waltham, MA, USA) and then reverse transcribed to cDNA using the Revert Aid First-strand cDNA Synthesis kit (Thermo Fisher Scientific, Waltham, MA, USA). The qRT-PCR was conducted using the SYBR Green Premix kit (Takara, Dalian, China) and the PikoReal 96 Real-Time PCR system (Thermo Scientific, Vantaa, Finland) following the manufacturer’s instructions. Target gene expression levels were evaluated using the 2^−ΔΔCt^ method [37]. The primers used to amplify target genes are listed in Table S1. The β-actin gene was selected as the reference gene.

### 4.8. Pathogenicity Assay

Pathogenicity tests were completed using maize seeds [38]. Embryos were removed to prevent germination, and endosperms were wounded by toothpicks to provide an infection site. Wounded kernels were surface-sterilized with 0.05% sodium hypochlorite for 3 min and 75% ethanol for 1 min. Sterilized seeds were rinsed three times with sterile water, and then added to 50-mL sterilized flasks. The seeds were inoculated with 10^7^ conidia, which were suspended in 15 mL sterile distilled water. After shaking at 180 rpm at 28 °C for 30 min, inoculated kernels were transferred to moistened filter paper in Petri dishes, and incubated at 28 °C. Kernels were harvested after 7 days to count spores and quantify aflatoxins.

## Supplementary Materials

The following are available online at www.mdpi.com/2072-6651/8/7/202/s1, Figure S1: The alignment between the sequenced products and the expected sequences. The *skn7-5* and *skn7-11* indicated the sequenced products, while the *pyrG* indicated the expected sequences. Sequences marked with red and yellow letters were two primer pairs (i.e., AflSkn7DR and AflSkn7UF, respectively), which were used for amplifying fusion fragments. Figure S2: Thin-layer chromatography analysis for aflatoxin production in the indicated strains. Each strain was inoculated into 25 mL of PDB broth (supplemented with Zinc and Molybdenum ions) in a 100 mL flask and incubated with shaking at 180 r/m at 28 °C. After 6 days, the aflatoxin was extracted using a chloroform method. ST, aflatoxin B1; WT, the wild type; Δ*Skn7-5* and Δ*Skn7-11*, two *AflSkn7* deletion mutants. Table S1: Primers used in this study.

## References

[1] Amaike S., Keller N.P. (2011). Aspergillus flavus. Annu. Rev. Phytopathol..

[2] Razzaghi-Abyaneh M., Chang P.-K., Shams-Ghahfarokhi M., Rai M. (2014). Global health issues of aflatoxins in food and agriculture: Challenges and opportunities. Front. Microbiol..

[3] Marin S., Ramos A., Cano-Sancho G., Sanchis V. (2013). Mycotoxins: Occurrence, toxicology, and exposure assessment. Food. Chem. Toxicol..

[4] Yu J., Chang P.-K., Ehrlich K.C., Cary J.W., Bhatnagar D., Cleveland T.E., Payne G.A., Linz J.E., Woloshuk C.P., Bennett J.W. (2004). Clustered pathway genes in aflatoxin biosynthesis. Appl. Environ. Microbiol..

[5] Georgianna D.R., Payne G.A. (2009). Genetic regulation of aflatoxin biosynthesis: From gene to genome. Fungal Genet. Biol..

[6] Grintzalis K., Vernardis S.I., Klapa M.I., Georgiou C.D. (2014). Role of oxidative stress in Sclerotial differentiation and aflatoxin B1 biosynthesis in *Aspergillus flavus*. Appl. Environ. Microbiol..

[7] Zhang F., Guo Z., Zhong H., Wang S., Yang W., Liu Y., Wang S. (2014). RNA-Seq-based transcriptome analysis of aflatoxigenic Aspergillus flavus in response to water activity. Toxins.

[8] Zhang F., Zhong H., Han X., Guo Z., Yang W., Liu Y., Yang K., Zhuang Z., Wang S. (2015). Proteomic profile of Aspergillus flavus in response to water activity. Fungal Biol..

[9] Bai Y., Wang S., Zhong H., Yang Q., Zhang F., Zhuang Z., Yuan J., Nie X., Wang S. (2015). Integrative analyses reveal transcriptome-proteome correlation in biological pathways and secondary metabolism clusters in, A. flavus in response to temperature. Sci. Rep..

[10] Fassler J.S., West A.H. (2011). Fungal Skn7 stress responses and their relationship to virulence. Eukaryot. Cell.

[11] Shalaby S., Larkov O., Lamdan N.L., Horwitz B.A. (2014). Genetic interaction of the stress response factors ChAP1 and Skn7 in the maize pathogen *Cochliobolus heterostrophus*. FEMS Microbiol. Lett..

[12] Mulford K., Fassler J. (2011). Association of the Skn7 and Yap1 transcription factors in the *Saccharomyces cerevisiae* oxidative stress response. Eukaryot. Cell.

[13] Yang Q., Yin D., Yin Y., Cao Y., Ma Z. (2015). The response regulator BcSkn7 is required for vegetative differentiation and adaptation to oxidative and osmotic stresses in *Botrytis cinerea*. Mol. Plant Pathol..

[14] Shang Y., Chen P., Chen Y., Lu Y., Wang C. (2015). MrSkn7 Controls Sporulation, Cell Wall Integrity, Autolysis, and Virulence in *Metarhizium robertsii*. Eukaryot. Cell.

[15] Hong S.-Y., Roze L.V., Linz J.E. (2013). Oxidative stress-related transcription factors in the regulation of secondary metabolism. Toxins.

[16] Vargas-Pérez I., Sánchez O., Kawasaki L., Georgellis D., Aguirre J. (2007). Response regulators SrrA and SskA are central components of a phosphorelay system involved in stress signal transduction and asexual sporulation in *Aspergillus nidulans*. Eukaryot. Cell.

[17] Hagiwara D., Mizuno T., Abe K. (2011). Characterization of the conserved phosphorylation site in the *Aspergillus nidulans* response regulator SrrA. Curr. Genet..

[18] Chen L.-H., Lin C.-H., Chung K.-R. (2012). Roles for SKN7 response regulator in stress resistance, conidiation and virulence in the citrus pathogen *Alternaria alternata*. Fungal Genet. Biol..

[19] Lamarre C., Ibrahim-Granet O., Du C., Calderone R., Latgé J.-P. (2007). Characterization of the SKN7 ortholog of *Aspergillus fumigatus*. Fungal Genet. Biol..

[20] Jiang C., Zhang S., Zhang Q., Tao Y., Wang C., Xu J.R. (2015). FgSKN7 and FgATF1 have overlapping functions in ascosporogenesis, pathogenesis and stress responses in *Fusarium graminearum*. Environ. Microbiol..

[21] Bahn Y.-S., Kojima K., Cox G.M., Heitman J. (2006). A unique fungal two-component system regulates stress responses, drug sensitivity, sexual development, and virulence of *Cryptococcus neoformans*. Mol. Biol. Cell.

[22] Viefhues A., Schlathoelter I., Simon A., Viaud M., Tudzynski P. (2015). Unraveling the function of the response regulator BcSkn7 in the stress signaling network of *Botrytis cinerea*. Eukaryot. Cell.

[23] Ohmiya R., Yamada H., Kato C., Aiba H., Mizuno T. (2000). The Prr1 response regulator is essential for transcription of *ste11*^+^ and for sexual development in fission yeast. Mol. Gen. Genet..

[24] Wei H., Requena N., Fischer R. (2003). The MAPKK kinase SteC regulates conidiophore morphology and is essential for heterokaryon formation and sexual development in the homothallic fungus *Aspergillus nidulans*. Mol. Microbiol..

[25] Morgan B.A., Banks G.R., Toone W.M., Raitt D., Kuge S., Johnston L.H. (1997). The Skn7 response regulator controls gene expression in the oxidative stress response of the budding yeast *Saccharomyces cerevisiae*. EMBO J..

[26] Motoyama T., Ochiai N., Morita M., Iida Y., Usami R., Kudo T. (2008). Involvement of putative response regulator genes of the rice blast fungus *Magnaporthe oryzae* in osmotic stress response, fungicide action, and pathogenicity. Curr. Genet..

[27] Levin D.E. (2011). Regulation of cell wall biogenesis in *Saccharomyces cerevisiae*: The cell wall integrity signaling pathway. Genetics.

[28] Roze L.V., Laivenieks M., Hong S.Y., Wee J., Wong S.S., Vanos B., Awad D., Ehrlich K.C., Linz J.E. (2015). Aflatoxin biosynthesis is a novel source of reactive oxygen species—A potential redox signal to initiate resistance to oxidative stress?. Toxins.

[29] Reverberi M., Zjalic S., Ricelli A., Punelli F., Camera E., Fabbri C., Picardo M., Fanelli C., Fabbri A.A. (2008). Modulation of antioxidant defense in *Aspergillus parasiticus* is involved in aflatoxin biosynthesis: A role for the Ap*yapA* gene. Eukaryot. Cell.

[30] Reverberi M., Gazzetti K., Punelli F., Scarpari M., Zjalic S., Ricelli A., Fabbri A.A., Fanelli C. (2012). Ao*yap1* regulates OTA synthesis by controlling cell redox balance in *Aspergillus ochraceus*. Appl. Microbial. Biotechnol..

[31] Hong S.-Y., Roze L.V., Wee J., Linz J.E. (2013). Evidence that a transcription factor regulatory network coordinates oxidative stress response and secondary metabolism in aspergilli. Microbiologyopen.

[32] Heller J., Tudzynski P. (2011). Reactive oxygen species in phytopathogenic fungi: Signaling, development, and disease. Annu. Rev. Phytopathol..

[33] Aspergillus Genome Projects. http://www.broadinstitute.org/annotation/genome.

[34] Simple Modular Architecture Research Tool. http://smart.embl-heidelberg.de/.

[35] Illustrator for Biological Sequences program. http://ibs.biocuckoo.org/.

[36] Szewczyk E., Nayak T., Oakley C.E., Edgerton H., Xiong Y., Taheri-Talesh N., Osmani S.A., Oakley B.R. (2006). Fusion PCR and gene targeting in *Aspergillus nidulans*. Nat. Protoc..

[37] Livak K.J., Schmittgen T.D. (2001). Analysis of relative gene expression data using real-time quantitative PCR and the 2^−ΔΔCT^ method. Methods.

[38] Gao X., Brodhagen M., Isakeit T., Brown S.H., Göbel C., Betran J., Feussner I., Keller N.P., Kolomiets M.V. (2009). Inactivation of the lipoxygenase ZmLOX3 increases susceptibility of maize to *Aspergillus* spp.. Mol. Plant Microbe Interact..

