# Supplementary material for: The Stress Response Regulator AflSkn7 Influences Morphological Development, Stress Response, and Pathogenicity in the Fungus Aspergillus flavus
Source: Toxins (Basel). 2016 Jul 5;8(7):202. doi: 10.3390/toxins8070202 (PMC4963835; doi:10.3390/toxins8070202)
Supplement: Supplementary file 1 [file toxins-08-00202-s001.pdf]

Supplementary Materials: The Stress Response Regulator AflSkn7 Influences Morphological Development, Stress Response, and Pathogenicity in the Fungus *Aspergillus flavus*

Feng Zhang, Gaopo Xu, Longpo Geng, Xiaoyan Lu, Kunlong Yang, Jun Yuan, Xinyi Nie, Zhenhong Zhuang and Shihua Wang

Table S1. Primers used in this study.

Primers	Sequences (5′-3′)
AflSkn7UF	CAGCAGCCTATCTCCATCTT
AflSkn7UR	GGGTGAAGAGCATTGTTTGAGGCCGAACAAGCAACAGGCTCAA
AflSkn7DF	GCATCAGTGCCTCCTCTCAGACACACGCAACCATTCACCTAC
AflSkn7DR	AACATCCGTCCTCGCATCAA
AflSkn7UUF	ATCAGTATGTGCCTTCCGAGTG
AflSkn7DDR	CGACGATAGCGATCTCCATT
pyrGF	GCCTCAAACAATGCTCTTCACCC
pyrGR	GTCTGAGAGGAGGCACTGATGC
AflSkn7F	TGTCGTCTTGGAGGTAATGC
AflSkn7R	ATTGTCTGCGAACTGTCTCC
AflSkn7RTF	CGGGCGAACAGCAAAGAATC
AflSkn7RTR	TCAAACTGCCGAATGAGGTG
AflJ/qF	CGTCTACACCTCTTGTCTTCTG
AflJ/qR	TCATCAACCTGGCATCATCTC
AflQ/qF	GTCGCATATGCCCCGGTCGG
AflQ/qR	GGCAACCAGTCGGGTTCCGG
NsdC/qF	CTTCATCGCGCTCACTCTCC
NsdC/qR	GGTTGCTAGAATGGCTGTGG
NsdD/qF	CGGAACCAGGATGCCTTACTAC
NsdD/qR	CTGCTTTGCCTCGTCGCTTC
gtr/qF	ACATTCTCGGACTCGGCGT
gtr/qR	AGCACTGGTAGGGTGGATCG
sod/qF	TGAGCGTGCCTTCCATGTC
sod/qR	CGTGGCGGTTCTCATCCTC
fsk1/qF	CAACATCGCCTGGTGGACT
fsk1/qR	CTTGCGGAGCTTAGACTGC
och1/qF	CACGGAGGAAACACTACGGATGAAGAAGG
och1/qR	CAGGGCTGAAGCTCGTAATGGGTAGAACT
Actin/qF	ACGGTGTCGTCACAAACTGG
Actin/qR	CGGTTGGACTTAGGGTTGATAG

```
pyrG         1 ------------------------------------------------------------------------------------------
skn7-11      1 --------------------------------GTATCGCCGTGAATGCGTATGAGAGGAGAGGGCGTCATCCA
skn7-5       1 CTGCGTTGGTCGGAGTTTAGTATCGCCGTGAATGCGTATGAGAGGAGAGGGCGTCATCCA

pyrG         1 ------------------------------------------------------------------------------------------
skn7-11     42 CGTACTTGATATACTTCTCCAGCTGATCCGAGTGTGCTCCGCCGTACAGGAACACGTCTT
skn7-5      61 CGTACTTGATATACTTCTCCAGCTGATCCGAGTGTGCTCCGCCGTACAGGAACACGTCTT

pyrG         1 ------------------------------------------------------------------------------------------
skn7-11    102 CGACACCGGCATTCGCAAGGAACTCCAATGTATACTCAATCAAGGGGGTGTTCGCTAGCG
skn7-5     121 CGACACCGGCATTCGCAAGGAACTCCAATGTATACTCAATCAAGGGGGTGTTCGCTAGCG

pyrG         1 ------------------------------------------------------------------------------------------
skn7-11    162 GCAAAAGACACTGTCATCGTCTGTGTCAGCCCATAACCCATTATTGTGTTTTCCCCGCAA
skn7-5     181 GCAAAAGACACTGTCATCGTCTGTGTCAGCCCATAACCCATTATTGTGTTTTCCCCGCAA

pyrG         1 ------------------------------------------------------------------------------------------
skn7-11    222 CATCCGTCCTCGCATCAATAGCTTGTCTTAGATACATACCCGGGGCTTGTCAAGAGTGAA
skn7-5     241 CATCCGTCCTCGCATCAATAGCTTGTCTTAGATACATACCCGGGGCTTGTCAAGAGTGAA

pyrG         1 ------------------------------------------------------------------------------------------
skn7-11    282 GGGTTCAAATCTCGTCTCGAAGGTGTCGGCAAGAACCTAAACAACATCAAAGCATTAGCC
skn7-5     301 GGGTTCAAATCTCGTCTCGAAGGTGTCGGCAAGAACCTAAACAACATCAAAGCATTAGCC

pyrG         1 ------------------------------------------------------------------------------------------
skn7-11    342 ACACGATCATCGTGCACCCCGCTGTCCTATGATGGCCAGCAGTCGCAAGAGTGGTTTTCC
skn7-5     361 ACACGATCATCGTGCACCCCGCTGTCCTATGATGGCCAGCAGTCGCAAGAGTGGTTTTCC

pyrG         1 ------------------------------------------------------------
skn7-11    402 AGAATGACAACATACGACAGCCTGCAACGTCTCCTCTACTTCCTCAGCAGCATTGCCCCT
skn7-5     421 AGAATGACAACATACGACAGCCTGCAACGTCTCCTCTACTTCCTCAGCAGCATTGCCCCT
consensus  421 ............................................................

pyrG         1 ------------------------------------------------------------
skn7-11    462 CTGCTTGGAGCCACCGCCTTTCTGCTTCGGACCCATCTGCTAAATCAATGGTAAGCGTGA
skn7-5     481 CTGCTTGGAGCCACCGCCTTTCTGCTTCGGACCCATCTGCTAAATCAATGGTAAGCGTGA

pyrG         1 ------------------------------------------------------------
skn7-11    522 GTTGCGCGGAAAGATCAAATGCCACATACTGCGACTCAATGAGTAACTAATTTCCCCCAC
skn7-5     541 GTTGCGCGGAAAGATCAAATGCCACATACTGCGACTCAATGAGTAACTAATTTCCCCCAC

pyrG         1 ------------------------------------------------------------
skn7-11    582 CTCACTGAAAGGTCAAATATTCCTGCAAAAAGTTTAGCAAGAGCGGATGTATTGTGGTAA
skn7-5     601 CTCACTGAAAGGTCAAATATTCCTGCAAAAAGTTTAGCAAGAGCGGATGTATTGTGGTAA

pyrG         1 ------------------------------------------------------------
skn7-11    642 AGGAAGCCCAGGCGATAAACGGGCTCACAAAGAAACCGAAAACCAACAATAAATCCGCTT
skn7-5     661 AGGAAGCCCAGGCGATAAACGGGCTCACAAAGAAACCGAAAACCAACAATAAATCCGCTT

pyrG         1 ------------------------------------------------------------
skn7-11    702 GCACAACCTAAAGCAATTATTTCCGGTTCCCCTCGACGCAAAAACTCCGCCAAGCCATAT
skn7-5     721 GCACAACCTAAAGCAATTATTTCCGGTTCCCCTCGACGCAAAAACTCCGCCAAGCCATAT
```

```
pyrG        1 ------------------------------------------------------------
skn7-11   762 TGAATGATTCATCCCACTCCCGTCCTTTTGTTTGCATGTTTGATCTGTTTGCGAGGTTTG
skn7-5    781 TGAATGATTCATCCCACTCCCGTCCTTTTGTTTGCATGTTTGATCTGTTTGCGAGGTTTG

pyrG        1 ------------------------------------------------------------
skn7-11   822 CCGCCCAAGTTCTGTTCCTTTCACGTGACCCACGTGACTGGGAGCATTAGCCACACACAA
skn7-5    841 CCGCCCAAGTTCTGTTCCTTTCACGTGACCCACGTGACTGGGAGCATTAGCCACACACAA

pyrG        1 ------------------------------------------------------------
skn7-11   882 CCTGAAGGACCAGCTTCAGCCCCAAAGAGTCCAGAACACCATCCAAGGCCCAGGCCCTCC
skn7-5    901 CCTGAAGGACCAGCTTCAGCCCCAAAGAGTCCAGAACACCATCCAAGGCCCAGGCCCTCC

pyrG        1 ------------------------------------------------------------
skn7-11   942 TGGACTGCTGGTAGTATCCCTTGTTCAGGAACCAAAGGTCTAGAACCTTAATATGCATCT
skn7-5    961 TGGACTGCTGGTAGTATCCCTTGTTCAGGAACCAAAGGTCTAGAACCTTAATATGCATCT

pyrG        1 ------------------------------------------------------------
skn7-11  1002 CTATTGTACGTCAAATTAAATAGGAGTTCCGGTCAGACGATATCTTCCCCCCGAGTCGAA
skn7-5   1021 CTATTGTACGTCAAATTAAATAGGAGTTCCGGTCAGACGATATCTTCCCCCCGAGTCGAA

pyrG        1 ------------------------------------------------------------
skn7-11  1062 GGGCAATCGGAAGCTCCCAAGCTCCTGTGGATTACCCGGTCCGTCTGGGGCTGGGGTAGT
skn7-5   1081 GGGCAATCGGAAGCTCCCAAGCTCCTGTGGATTACCCGGTCCGTCTGGGGCTGGGGTAGT

pyrG        1 ------------------------------------------------------------
skn7-11  1122 GCAAAGAGTGTGGAATGATATGTGTTCCGGACGAAGCATACCCACTTGTGTACGTGTGCG
skn7-5   1141 GCAAAGAGTGTGGAATGATATGTGTTCCGGACGAAGCATACCCACTTGTGTACGTGTGCG

pyrG        1 ------------------------------------------------------------
skn7-11  1182 CACAGTGTAGGAAGGGCTTCTGAGTGGAGGATATATTGCAGGATGACTGATCTGAAGAAT
skn7-5   1201 CACAGTGTAGGAAGGGCTTCTGAGTGGAGGATATATTGCAGGATGACTGATCTGAAGAAT

pyrG        1 ---------------------GTCTGAGAGGAGGCACTGATGCGTGATGCCAAGCTGTTA
skn7-11  1242 GGTAGGTGAATGGTTGCGTGTGTCTGAGAGGAGGCACTGATGCGTGATGCCAAGCTGTTA
skn7-5   1261 GGTAGGTGAATGGTTGCGTGTGTCTGAGAGGAGGCACTGATGCGTGATGCCAAGCTGTTA

pyrG       40 TCTCTTCTGCACAGGTCGACTTGTCCTGAGGGACCTTCCATATTAAGCTATCAGTCCACT
skn7-11  1302 TCTCTTCTGCACAGGTCGACTTGTCCTGAGGGACCTTCCATATTAAGCTATCAGTCCACT
skn7-5   1321 TCTCTTCTGCACAGGTCGACTTGTCCTGAGGGACCTTCCATATTAAGCTATCAGTCCACT

pyrG      100 TTGCTTATCGACTACATAGATAAAGCCTTGGCGTTTCTGGCAAAGCTTACCTCACTTGCT
skn7-11  1362 TTGCTTATCGACTACATAGATAAAGCCTTGGCGTTTCTGGCAAAGCTTACCTCACTTGCT
skn7-5   1381 TTGCTTATCGACTACATAGATAAAGCCTTGGCGTTTCTGGCAAAGCTTACCTCACTTGCT

pyrG      160 AGATGACTGGTAGGAATCTAACAAAGGGAAAACCTTGACGATTGGGGGCGGAAAACATGG
skn7-11  1422 AGATGACTGGTAGGAATCTAACAAAGGGAAAACCTTGACGATTGGGGGCGGAAAACATGG
skn7-5   1441 AGATGACTGGTAGGAATCTAACAAAGGGAAAACCTTGACGATTGGGGGCGGAAAACATGG

pyrG      220 CATTACAGGGGCCAATACGGACGAGCTCATCGTTTAATGACAGGCACAATGTATGCAGCC
skn7-11  1482 CATTACAGGGGCCAATACGGACGAGCTCATCGTTTAATGACAGGCACAATGTATGCAGCC
skn7-5   1501 CATTACAGGGGCCAATACGGACGAGCTCATCGTTTAATGACAGGCACAATGTATGCAGCC

pyrG      280 TTCCTCCACAACACTCGTACTGGCACTACACTTTTGCTCCAAGAGGAAATCATGACTTGC
skn7-11  1542 TTCCTCCACAACACTCGTACTGGCACTACACTTTTGCTCCAAGAGGAAATCATGACTTGC
skn7-5   1561 TTCCTCCACAACACTCGTACTGGCACTACACTTTTGCTCCAAGAGGAAATCATGACTTGC
```

```
pyrG      340  CGCATACTCTGGCCATATAAGCTTCCCAGCCTTCTTTCTGGTACCGCTGTGCAGCTTCAA
skn7-11   1602 CGCATACTCTGGCCATATAAGCTTCCCAGCCTTCTTTCTGGTACCGCTGTGCAGCTTCAA
skn7-5    1621 CGCATACTCTGGCCATATAAGCTTCCCAGCCTTCTTTCTGGTACCGCTGTGCAGCTTCAA

pyrG      400  CCGGGTCGGGAGCAGCGTAGATGCCTCGACCGGCGATGATAAAGTCGGCACCGCGTCCAA
skn7-11   1662 CCGGGTCGGGAGCAGCGTAGATGCCTCGACCGGCGATGATAAAGTCGGCACCGCGTCCAA
skn7-5    1681 CCGGGTCGGGAGCAGCGTAGATGCCTCGACCGGCGATGATAAAGTCGGCACCGCGTCCAA

pyrG      460  TAGCCGATGCAGGAGTCTGGTATTGCTGTCCAAGCTTATCTCCTTTGGAAGAGAGGTTCA
skn7-11   1722 TAGCCGATGCAGGAGTCTGGTATTGCTGTCCAAGCTTATCTCCTTTGGAAGAGAGGTTCA
skn7-5    1741 TAGCCGATGCAGGAGTCTGGTATTGCTGTCCAAGCTTATCTCCTTTGGAAGAGAGGTTC
pyrG      520  CACCCGTCGTGAAGACCACGAAATCTTCATCCTCCGAGGCTGAAGACACATCCGACTGCA
skn7-11   1782 CACCCGTCGTGAAGACCACGAAATCTTCATCCTCCGAGGCTGAAGACACATCCGACTGCA
skn7-5    1801 CACCCGTCGTGAAGACCACGAAATCTTCATCCTCCGAGGCTGAAGACACATCCGACTGCA

pyrG      580  CTTCCGTCAGGGCCCGCGTCGACACGAAACCCATAACGAAGTTCTTGTATTTGCGAGCGT
skn7-11   1842 CTTCCGTCAGGGCCCGCGTCGACACGAAACCCATAACGAAGTTCTTGTATTTGCGAGCGT
skn7-5    1861 CTTCCGTCAGGGCCCGCGTCGACACGAAACCCATAACGAAGTTCTTGTATTTGCGAGCGT
pyrG      640  AGTCAACCGATGCCTTGGTATACTCGCCCGTAGCCAGCGATCCTTTGGAGGTCATCTCTG
skn7-11   1902 AGTCAACCGATGCCTTGGTATACTCGCCCGTAGCCAGCGATCCTTTGGAGGTCATCTCTG
skn7-5    1921 AGTCAACCGATGCCTTGGTATACTCGCCCGTAGCCAGCGATCCTTTGGAGGTCATCTCTG

pyrG      700  CCAGGACCAACAGTCCTCTCTCAGGACCATAGGGGAAGTCTTGCGCAGATGCGGTCTGGG
skn7-11   1962 CCAGGACCAACAGTCCTCTCTCAGGACCATAGGGGAAGTCTTGCGCAGATGCGGTCTGGG
skn7-5    1981 CCAGGACCAACAGTCCTCTCTCAGGACCATAGGGGAAGTCTTGCGCAGATGCGGTCTGGG

pyrG      760  CCAGAGCCTCGACGATGCCCTCGCCAGGGAGAACGCTGCAGTTGATAATGTGGGCCCATT
skn7-11   2022 CCAGAGCCTCGACGATGCCCTCGCCAGGGAGAACGCTGCAGTTGATAATGTGGGCCCATT
skn7-5    2041 CCAGAGCCTCGACGATGCCCTCGCCAGGGAGAACGCTGCAGTTGATAATGTGGGCCCATT

pyrG      820  CGGAGATCCTCAGAGCACCGCCGTGGTATTGCTTCTGGACGGTATTGCCGATGTCGATGA
skn7-11   2082 CGGAGATCCTCAGAGCACCGCCGTGGTATTGCTTCTGGACGGTATTGCCGATGTCGATGA
skn7-5    2101 CGGAGATCCTCAGAGCACCGCCGTGGTATTGCTTCTGGACGGTATTGCCGATGTCGATGA

pyrG      880  ATTTGCGGTCCTCGAAGATCAAAAAGTTGTGCTTTTGAGCCAGCACATTCAGGCCATTGA
skn7-11   2142 ATTTGCGGTCCTCGAAGATCAAAAAGTTGTGCTTTTGAGCCAGCACATTCAGGCCATTGA
skn7-5    2161 ATTTGCGGTCCTCGAAGATCAAAAAGTTGTGCTTTTGAGCCAGCACATTCAGGCCATTGA
pyrG      940  TAGTGTCGACGCTGAAATCGGTGAGGATGTCGATGTGTGTCTTGATGACGGCGATGTAGG
skn7-11   2202 TAGTGTCGACGCTGAAATCGGTGAGGATGTCGATGTGTGTCTTGATGACGGCGATGTAGG
skn7-5    2221 TAGTGTCGACGCTGAAATCGGTGAGGATGTCGATGTGTGTCTTGATGACGGCGATGTAGG

pyrG      1000 GACCGAGACCTGTATCATCAAAAGTTGATTAGTCCATTGCTAGACGGCATATGTATTGGA
skn7-11   2262 GACCGAGACCTGTATCATCAAAAGTTGATTAGTCCATTGCTAGACGGCATATGTATTGGA
skn7-5    2281 GACCGAGACCTGTATCATCAAAAGTTGATTAGTCCATTGCTAGACGGCATATGTATTGGA

pyrG      1060 TCCAACAGCTTCCGTACGGTCAGCGAGGTCCAGGAGTTCTCGGGTTGTCGTCACATCAGC
skn7-11   2322 TCCAACAGCTTCCGTACGGTCAGCGAGGTCCAGGAGTTCTCGGGTTGTCGTCACATCAGC
skn7-5    2341 TCCAACAGCTTCCGTACGGTCAGCGAGGTCCAGGAGTTCTCGGGTTGTCGTCACATCAGC

pyrG      1120 AGAGACGGTAACGTTTGTCTTCTTTGCTTCGGCAATCTCAAAAAGTCTCTTTGCCAGAGG
skn7-11   2382 AGAGACGGTAACGTTTGTCTTCTTTGCTTCGGCAATCTCAAAAAGTCTCTTTGCCAGAGG
skn7-5    2401 AGAGACGGTAACGTTTGTCTTCTTTGCTTCGGCAATCTCAAAAAGTCTCTTTGCCAGAGG
```

```
pyrG      1180 ATTGGGGTGCTTGCTGGCTCGAGCACCGTAAGTCAATTGCGACTTGGACGACATCGTGGG
skn7-11   2442 ATTGGGGTGCTTGCTGGCTCGAGCACCGTAAGTCAATTGCGACTTGGACGACATCGTGGG
skn7-5    2461 ATTGGGGTGCTTGCTGGCTCGAGCACCGTAAGTCAATTGCGACTTGGACGACATCGTGGG

pyrG      1240 AATGGAGGGTTATATGCGGGGTATGACGGTGATTGATGAGTTGAAGTAGCCGAGCAATGA
skn7-11   2502 AATGGAGGGTTATATGCGGGGTATGACGGTGATTGATGAGTTGAAGTAGCCGAGCAATGA
skn7-5    2521 AATGGAGGGTTATATGCGGGGTATGACGGTGATTGATGAGTTGAAGTAGCCGAGCAATGA

pyrG      1300 GGTATATTATCCAATTGAGGGTGACCCAACCAAATCAATTGCTTGAGAATCAAATCTCAA
skn7-11   2562 GGTATATTATCCAATTGAGGGTGACCCAACCAAATCAATTGCTTGAGAATCAAATCTCAA
skn7-5    2581 GGTATATTATCCAATTGAGGGTGACCCAACCAAATCAATTGCTTGAGAATCAAATCTCAA

pyrG      1360 ATATTCTAAAATAGAAGCCGCCGGGGACTAAATTTAAAGTCGTACTCTATCTCGATCTTT
skn7-11   2622 ATATTCTAAAATAGAAGCCGCCGGGGACTAAATTTAAAGTCGTACTCTATCTCGATCTTT
skn7-5    2641 ATATTCTAAAATAGAAGCCGCCGGGGACTAAATTTAAAGTCGTACTCTATCTCGATCTTT

pyrG      1420 TCCGCGTGTAAAAATTGCGTGCCAATCTGGATGGAGACAGGCCACATCGGTGCTGTATTC
skn7-11   2682 TCCGCGTGTAAAAATTGCGTGCCAATCTGGATGGAGACAGGCCACATCGGTGCTGTATTC
skn7-5    2701 TCCGCGTGTAAAAATTGCGTGCCAATCTGGATGGAGACAGGCCACATCGGTGCTGTATTC

pyrG      1480 CTCCGCCTCCGACTGTGGGGTCAACCACAATTAAACTATTCTGTAGTCAGGTACAGCTAG
skn7-11   2742 CTCCGCCTCCGACTGTGGGGTCAACCACAATTAAACTATTCTGTAGTCAGGTACAGCTAG
skn7-5    2761 CTCCGCCTCCGACTGTGGGGTCAACCACAATTAAACTATTCTGTAGTCAGGTACAGCTAG

pyrG      1540 AATGGGGTAGACAGGCAGAACTGTAGAAGATAAAACATTGGTCAATCACTGGTAACTCCA
skn7-11   2802 AATGGGGTAGACAGGCAGAACTGTAGAAGATAAAACATTGGTCAATCACTGGTAACTCCA
skn7-5    2821 AATGGGGTAGACAGGCAGAACTGTAGAAGATAAAACATTGGTCAATCACTGGTAACTCCA

pyrG      1600 CGGAACTTTTAACGTACATATATATATATACACAACATATTTCGTCAGACACAGAATAAC
skn7-11   2862 CGGAACTTTTAACGTACATATATATATATACACAACATATTTCGTCAGACACAGAATAAC
skn7-5    2881 CGGAACTTTTAACGTACATATATATATATACACAACATATTTCGTCAGACACAGAATAAC

pyrG      1660 TCTCAGTAAGAGATCATGTCTTTGGCAACACCAAACACACTCAGAGCCCACAGAGCGCCT
skn7-11   2922 TCTCAGTAAGAGATCATGTCTTTGGCAACACCAAACACACTCAGAGCCCACAGAGCGCCT
skn7-5    2941 TCTCAGTAAGAGATCATGTCTTTGGCAACACCAAACACACTCAGAGCCCACAGAGCGCCT

pyrG      1720 TGAGAAAACCAGAAGAAACCTCCCAGCAATCTGCCCTTGCCTTGTGGCTTACCCGAGTAC
skn7-11   2982 TGAGAAAACCAGAAGAAACCTCCCAGCAATCTGCCCTTGCCTTGTGGCTTACCCGAGTAC
skn7-5    3001 TGAGAAAACCAGAAGAAACCTCCCAGCAATCTGCCCTTGCCTTGTGGCTTACCCGAGTAC

pyrG      1780 ACATAGCCGTACAAGTAGAGAGATCGGAAAAACAGCCATGAAGCGCCAATTGCTGTTGCC
skn7-11   3042 ACATAGCCGTACAAGTAGAGAGATCGGAAAAACAGCCATGAAGCGCCAATTGCTGTTGCC
skn7-5    3061 ACATAGCCGTACAAGTAGAGAGATCGGAAAAACAGCCATGAAGCGCCAATTGCTGTTGCC

pyrG      1840 AGGTGAGGGTATTTCAGACCCGCGAAGAGGGTGAAGAGCATTGTTTGAGGC---------
skn7-11   3102 AGGTGAGGGTATTTCAGACCCGCGAAGAGGGTGAAGAGCATTGTTTGAGGCCGAACAAGC
skn7-5    3121 AGGTGAGGGTATTTCAGACCCGCGAAGAGGGTGAAGAGCATTGTTTGAGGCCGAACAAGC

pyrG           ------------------------------------------------------------
skn7-11   3162 AACAGGCTCAAGCACGTCGCAGAACCTCAAGAACAACGGCTGCGGGAGAGTATGAAAAGA
skn7-5    3181 AACAGGCTCAAGCACGTCGCAGAACCTCAAGAACAACGGCTGCGGGAGAGTATGAAAAGA

pyrG           ------------------------------------------------------------
skn7-11   3222 ACAAGAAAATGAACATCCAAGTCGGTGGTCAATTACGCACCGGGATCGAGAAGGAGGAAT
skn7-5    3241 ACAAGAAAATGAACATCCAAGTCGGTGGTCAATTACGCACCGGGATCGAGAAGGAGGAAT
```

```
pyrG             ------------------------------------------------------------
skn7-11    3282  TTCCGGGAGAAAGGATAAGTAAGAATAACGGTGAAGAAGAGAAAGAGAAAAGAAAAAAAG
skn7-5     3301  TTCCGGGAGAAAGGATAAGTAAGAATAACGGTGAAGAAGAGAAAGAGAAAAGAAAAAAAG

pyrG             ------------------------------------------------------------
skn7-11    3342  AAGGGAAAAAAATGACGGTCTAACCTGGACGGCGTTTGGTTGGGAGAGGTCAAGAGAAAG
skn7-5     3361  AAGGGAAAAAAATGACGGTCTAACCTGGACGGCGTTTGGTTGGGAGAGGTCAAGAGAAAG

pyrG             ------------------------------------------------------------
skn7-11    3402  AGTAGGGAAAGAGATGGTGGCAGTTCCCTTCAGTTAAGTTTTGGGTAATTTACTACTTCT
skn7-5     3421  AGTAGGGAAAGAGATGGTGGCAGTTCCCTTCAGTTAAGTTTTGGGTAATTTACTACTTCT

pyrG             ------------------------------------------------------------
skn7-11    3462  TGCGAAAAGTGGGACTTTTGATGGGGGGTCACTGACTTGGTATCTGCACCCGCCATTGGC
skn7-5     3481  TGCGAAAAGTGGGACTTTTGATGGGGGGTCACTGACTTGGTATCTGCACCCGCCATTGGC

pyrG             ------------------------------------------------------------
skn7-11    3522  CTTTCCTGTGCTGTCCGAACCACCGGTCACCGTTCACTAGCCACAACTTGCCATTTTTCC
skn7-5     3541  CTTTCCTGTGCTGTCCGAACCACCGGTCACCGTTCACTAGCCACAACTTGCCATTTTTCC
pyrG             ------------------------------------------------------------
skn7-11    3582  TCTGCTTTTTTTTACTTTTCAAATTCCTTTCATTGCTTTTTCTTTTTTTTTTTTGGTCCT
skn7-5     3601  TCTGCTTTTTTTTACTTTTCAAATTCCTTTCATTGCTTTTTCTTTTTTTTTTTTGGTCCT

pyrG             ------------------------------------------------------------
skn7-11    3642  CCCCTTCTGAAATTCTTTGGTACACATTATGGCAATGTTAAACAGCTTTACCTCCGATAA
skn7-5     3661  CCCCTTCTGAAATTCTTTGGTACACATTATGGCAATGTTAAACAGCTTTACCTCCGATAA

pyrG             ------------------------------------------------------------
skn7-11    3702  TGCAAGGAGCTAAAAAGGGGAAGGGCAGAAGGCCAGGAGCTCCTCTCTGACACACTGACC
skn7-5     3721  TGCAAGGAGCTAAAAAGGGGAAGGGCAGAAGGCCAGGAGCTCCTCTCTGACACACTGACC

pyrG             ------------------------------------------------------------
skn7-11    3762  GTAAACAACAGCCGACAAGACATCTACTAAGTGCAATCACCTCGGAGGGGAAATGTCGAT
skn7-5     3781  GTAAACAACAGCCGACAAGACATCTACTAAGTGCAATCACCTCGGAGGGGAAATGTCGAT

pyrG             ------------------------------------------------------------
skn7-11    3822  GTGGAGACAACAATCCACTTCTCGTACTCTCTGCCTTAATGCCTTAAGAGTAAAGAACAT
skn7-5     3841  GTGGAGACAACAATCCACTTCTCGTACTCTCTGCCTTAATGCCTTAAGAGTAAAGAACAT
pyrG             ------------------------------------------------------------
skn7-11    3882  GGTTTGATCATTACGAGTAGATTTGTCGCCAGTCATGAAGGGACAGCTAATTACATCCGA
skn7-5     3901  GGTTTGATCATTACGAGTAGATTTGTCGCCAGTCATGAAGGGACAGCTAATTACATCCGA

pyrG             ------------------------------------------------------------
skn7-11    3942  GTTATTCTCTCTTTGCCTTTCGTCGTGTTCTGGCGTCCAAGGAGTGAACGTTGCAAAGAA
skn7-5     3961  GTTATTCTCTCTTTGCCTTTCGTCGTGTTCTGGCGTCCAAGGAGTGAACGTTGCAAAGAA

pyrG             ------------------------------------------------------------
skn7-11    4002  AGGTCAATGCGTTCATGATGCAGATAACGTGCGAGGAAAGGATGGAGGGGAAAAGATGGA
skn7-5     4021  AGGTCAATGCGTTCATGATGCAGATAACGTGCGAGGAAAGGATGGAGGGGAAAAGATGGA

pyrG             ------------------------------------------------------------
skn7-11    4062  GATAGGCTGCTGGAGATGCAAGAGGAGGGGCAAATCAACCTGCAGCGATCGTCGAACAAA
skn7-5     4081  GATAGGCTGCTGGAGATGCAAGAGGAGGGGCAAATCAACCTGCAGCGATCGTCGAACAAA
```

```
pyrG              ------------------------------------------------------------
skn7-11     4122 AAAAGAGGAGAGAATGAGCGGAGACTCTGTATTCTCATCGACATACTAGACAATCATGAC
skn7-5      4141 AAAAGAGGAGAGAATGAGCGGAGACTCTGTATTCTCATCGACATACTAGACAATCATGAC

pyrG              ------------------------------------------------------------
skn7-11     4182 ATTGACCCTTTTTTTTAATCCAATCATTCAACCATCCGAATTTTGAGGGCACTAGTCAAA
skn7-5      4201 ATTGACCCTTTTTTTTAATCCAATCATTCAACCATCCGAATTTTGAGGGCACTAGTCAAA

pyrG              ------------------------------------------------------------
skn7-11     4242 AAGGTGACCTTGAATCATTCCTTTTTAGGATGCATTCTAGTCAGGTGTACATATTACATA
skn7-5      4261 AAGGTGACCTTGAATCATTCCTTTTTAGGATGCATTCTAGTCAGGTGTACATATTACATA

pyrG              ------------------------------------------------------------
skn7-11     4302 ACATTAATTATGTACACACGGTGGTATGTACTAGTATGTACTATGTAGTACTCCTTTGCA
skn7-5      4321 ACATTAATTATGTACACACGGTGGTATGTACTAGTATGTACTATGTAGTACTCCTTTGCA
pyrG              ------------------------------------------------------------
skn7-11     4362 TGGATCCATACTTCACACTACTTATGTAGTAGGGTAGTATTGACTGAGAGAATTTTTATT
skn7-5      4381 TGGATCCATACTTCACACTACTTATGTAGTAGGGTAGTATTGACTGAGAGAATTTTTATT

pyrG              ------------------------------------------------------------
skn7-11     4422 TATTCATTTTCCCCCCTTGAAGATAGGAGACAAATAAAGATATCATTGACTTTCTTTGTC
skn7-5      4441 TATTCATTTTCCCCCCTTGAAGATAGGAGACAAATAAAGATATCATTGACTTTCTTTGTC

pyrG              ------------------------------------------------------------
skn7-11     4482 CTTCACGTTTCAGTTGTACAGTTGTTTAGTTTTAAATCCTCAACGGTGGAGACTA-----
skn7-5      4501 CTTCACGTTTCAGTTGTACAGTTGTTTAGTTTTAAATCCTCAACGGTGGAGACTACCCCA

pyrG              ----------------
skn7-11          ----------------
skn7-5      4561 ATTGTGTTTTGGTTCA
```

Figure S1. The alignment between the sequenced products and the expected sequences. The *skn7-5* and *skn7-11* indicated the sequenced products, while the *pyrG* indicated the expected sequences. Sequences marked with red and yellow letters were two primer pairs (i.e., Afl*Skn7*DR and Afl*Skn7*UF, respectively), which were used for amplifying fusion fragments.

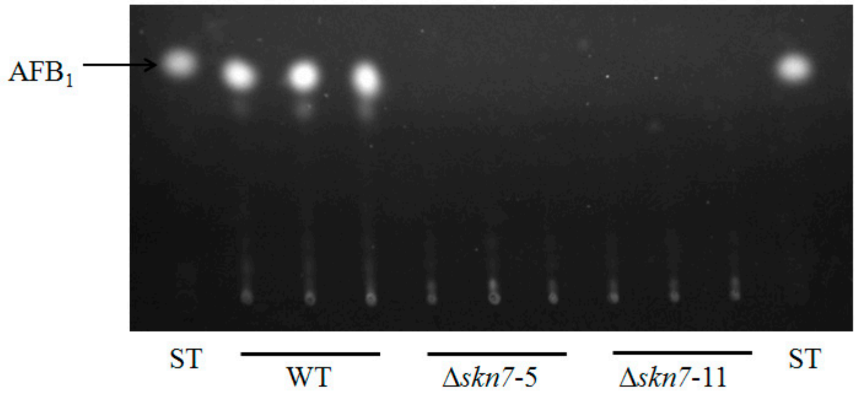

Figure S2. Thin-layer chromatography analysis for aflatoxin production in the indicated strains. Each strain was inoculated into 25 mL of potato dextrose broth (PDB) (supplemented with Zinc and Molybdenum ions) in a 100 mL flask and incubated with shaking at 180 r/m at 28 °C. After six days, the aflatoxin was extracted using a chloroform method. ST, aflatoxin B1; WT, the wild type; Δ*Skn7-5* and Δ*Skn7-11*, two *AflSkn7* deletion mutants.
